# Isotropic 3D compressed sensing (CS) based sequence is comparable to 2D-LGE in left ventricular scar quantification in different disease entities

**DOI:** 10.1007/s10554-022-02571-6

**Published:** 2022-03-03

**Authors:** Maximilian Fenski, Thomas Hiroshi Grandy, Darian Viezzer, Stela Kertusha, Michaela Schmidt, Christoph Forman, Jeanette Schulz-Menger

**Affiliations:** 1 Working Group Cardiovascular Magnetic Resonance, Experimental and Clinical Research Center, Charité Medical Faculty, Max-Delbrück Center for Molecular Medicine, Helios Klinikum Berlin Buch, Department of Cardiology and Nephrology, Charité – Universitätsmedizin Berlin, Kardiologie – ECRC, Lindenberger Weg 80, 13125 Berlin, Germany; 2 Siemens Healthineers, Erlangen, Germany; 3 DZHK (German Center for Cardiovascular Research), Partner Site Berlin, Berlin, Germany

**Keywords:** Late gadolinium enhancement, Myocardial scarring, Isotropic spatial resolution, Ischemic heart disease, Inflammatory heart disease, Compressed sensing

## Abstract

The goal of this study was to evaluate a three-dimensional compressed sensing (3D-CS) LGE prototype sequence for the detection and quantification of myocardial fibrosis in patients with chronic myocardial infarction (CMI) and myocarditis (MYC) compared with a 2D-LGE standard. Patients with left-ventricular LGE due to CMI (*n* = 33) or MYC (*n* = 20) were prospectively recruited. 2D-LGE and 3D-CS images were acquired in random order at 1.5 Tesla. 3D-CS short axis (SAX) images were reconstructed corresponding to 2D SAX images. LGE was quantitatively assessed on patient and segment level using semi-automated threshold methods. Image quality (4-point scoring system), Contrast-ratio (CR) and acquisition times were compared. There was no significant difference between 2D and 3D sequences regarding global LGE (%) (CMI [2D-LGE: 11.4 ± 7.5; 3D-LGE: 11.5 ± 8.5; *p* = 0.99]; MYC [2D-LGE: 27.0 ± 15.7; 3D-LGE: 26.2 ± 13.1; *p* = 0.70]) and segmental LGE-extent (*p* = 0.63). 3D-CS identified papillary infarction in 5 cases which was not present in 2D images. 2D-LGE acquisition time was shorter (2D: median: 06:59 min [IQR: 05:51–08:18]; 3D: 14:48 min [12:45–16:57]). 3D-CS obtained better quality scores (2D: 2.06 ± 0.56 vs. 3D: 2.29 ± 0.61). CR did not differ (*p* = 0.63) between basal and apical regions in 3D-CS images but decreased significantly in 2D apical images (CR basal: 2D: 0.77 ± 0.11, 3D: 0.59 ± 0.10; CR apical: 2D: 0.64 ± 0.17, 3D: 0.53 ± 0.11). 3D-LGE shows high congruency with standard LGE and allows better identification of small lesions. However, the current 3D-CS LGE sequence did not provide PSIR reconstruction and acquisition time was longer.

## Background

Late gadolinium enhancement (LGE) is a standard method for the assessment of focal myocardial fibrosis and scarring. It provides differentiation between ischemic and non-ischemic causes of myocardial injury [1], assessment of viability [2], and has gained in importance in cardiovascular risk stratification in a variety of diseases, e.g., myocardial infarction [3], dilated cardiomyopathy [4], hypertrophic cardiomyopathy (HCM) [5], or inflammatory heart disease (IHD) [6]. Consequentially, LGE assessment is an essential part of most contrast based CMR protocols and has been incorporated in recent clinical guidelines [7, 8].

Conventional two-dimensional (2D) breath-hold (bh) or motion corrected (MOCO) non-bh, phase sensitive inversion recovery (PSIR), electrocardiography (ECG)-gated, segmented spoiled gradient echo (GRE) readout based sequences are the standard sequence for LGE assessment [9]. Spatial resolution of these sequences typically amounts to 1.4–2.1 × 1.4–2.1 mm^2^ in-plane with a slice thickness of 6.0–8.0 mm [9, 10]. However, as spatial resolution exceeds the extent of small and thin-walled cardiac structures, e.g., the atria or right ventricle, small abnormalities in these structures may be missed. In addition, the application of anisotropic voxels can cause anatomical inaccuracies [11].

In order to address these issues, compressed sensing (CS) based, isotropic high resolution three-dimensional (3D)-LGE imaging has been introduced, allowing whole heart coverage with higher spatial resolution (typically 1.2^3^–1.7^3^ mm^3^) [12, 13]. CS is a recent approach for significantly reducing scan time by exploiting advanced reconstruction algorithms for sparsely sampled *k*-space data. It is based on an incoherent sub-sampling of the *k*-space, sparsity transformation and non-linear iterative reconstruction [14]. When combined with techniques to reduce respiratory artifacts, e.g., respiratory self-navigation or navigator gating, 3D-CS LGE can be obtained in free breathing technique, potentially further reducing scan time and increasing patient comfort while offering high spatial resolution [15].

Usage of isotropic voxel sizing can potentially reduce partial volume effects, which may improve tissue boundary delineation. Isotropic 3D-LGE is increasingly being used in scar assessment in patients with ischemic [15, 16] and non-ischemic [13] heart disease. Accurate and reliable fibrosis or scar assessment is an important prerequisite for precise risk stratification and optimized therapy planning in patients with myocardial infarction [17] and myocarditis [18]. However, prospective studies comparing isotropic high-resolution 3D-LGE with reference standard 2D-LGE for the quantitative assessment of left ventricular LGE in entities with different scar appearances are lacking so far. Therefore, we compared a non-bh 3D-CS prototype sequence with high isotropic spatial resolution (1.25^3^ mm^3^) to a 2D reference standard regarding image quality and accuracy of quantitative myocardial fibrosis assessment in the left ventricle (LV) in patients with chronic myocardial infarction (CMI) and myocarditis (MYC) in a prospective setting.

## Methods

### Study population

53 consecutive patients with CMI (*n* = 33) or MYC (*n* = 20) and suspicion for LGE on single-shot imaging performed as a backup set in our routine protocol, were prospectively recruited. Scans were performed between August 7th 2018 and December 4th, 2020. All patients were referred for LGE assessment based on clinical information provided by the referring cardiologist. Participants had to be in sinus rhythm. Exclusion criteria were contraindications to CMR, arrhythmia and severe chronic renal disease with an estimated glomerular filtration rate < 30 ml/min/1.73 m^2^.

### Data acquisition

CMR studies were performed on a 1.5 Tesla scanner (MAGNETOM AvantoFit®, Siemens Healthineers, Germany). Patients were scanned with ECG-gating in the supine position using 16-channel surface phased array coils. Imaging protocols for ischemic and non-ischemic heart disease included assessment of cardiac function in balanced steady-state free precession (bSSFP) cine sequences and of tissue characterization by LGE imaging. BSSFP cine imaging was performed in long axis 2- and 4-chamber view (CV) for biplanar assessment of LV end-diastolic volume (LVEDV), LV mass (LVM) and LV ejection fraction (LVEF). For LGE imaging, an intravenous contrast bolus of 0.2 mmol/kg gadoteridol (ProHance®, Bracco S.p.A., Milan, Italy) was administered. Two separate LGE sequences were acquired in random order: (1) Conventional 2D-LGE phase-sensitive inversion recovery (PSIR) sequence and (2) isotropic-resolution (1.25^3^ mm^3^) 3D-CS LGE.

### Two-dimensional LGE sequence

2D-LGE images were acquired with identical slice positioning to cine bSSFP images using a segmented single slice, single breath-hold 2D PSIR gradient-echo pulse sequence. A short-axis (SAX) stack covering both ventricles, 2-, 3-, and 4-CV was obtained with the following parameters: in-plane resolution 1.4 × 1.4 mm^2^, slice thickness 7 mm, no interslice gap, TE/TR/FA: 5.2 ms/10.2 ms/30°, matrix: 256 × 192, FOV: 350^2^–450^2^ mm^2^, parallel imaging with acceleration factor 2 was used. To determine optimal inversion time (TI) a segmented IR cine bSSFP TI scouting sequence was performed at a mid-ventricular short axis location before 2D-LGE sequence acquisition. Acquisition times for the 2D-LGE images, including SAX stack, 2-, 3- and 4-CV, were extracted from DICOM time stamps.

### Three-dimensional compressed sensing LGE sequence

The 3D inversion recovery prepared spoiled gradient-echo prototype sequence (Siemens WIP 1090) was acquired in free breathing, covering the whole heart in transverse orientation with the following parameters: TE/TR/FA: 2.4 ms/5.4 ms/15°, matrix: 256 × 248 × 88–112, acquired matrix-segments per R-R interval: 22, resulting scan window: 118.8 ms, isotropic resolution: 1.25^3^ mm^3^, FOV: 320 × 320 mm^2^. TI was individually adjusted using a TI Scout and 30 ms were added to account for time delay during 3D data acquisition. Navigator-based gating was used, positioned on the upper dome of the liver with a 6 mm (± 3 mm) acceptance window. The timing of data acquisition was adjusted manually to obtain readouts during the diastole. 4-CV cine images were used to identify the start time of the most quiescent window during ventricle diastole relative to the R wave. This time was used as the trigger delay.

### 3D-CS sampling pattern and reconstruction

Data acquisition was accelerated by sparse, incoherent sub-sampling of the phase-encoding plane, using a Cartesian variable-density spiral phyllotaxis pattern [19].

Motion-corrected under sampled 3D data were reconstructed using CS reconstruction based on 3D regularization using orthogonal Haar wavelets [19]. The cost function of the CS reconstruction was solved with a fast iterative shrinkage-thresholding algorithm (FISTA) [20]. External reference lines were used to estimate coil sensitivity maps (ESPIRiT) [21]. Reference scans for the estimation of the coil sensitivity maps were acquired separately from the image data at the beginning of the scan. The actual acceleration factor was 5.1 compared to the fully sampled *k*-space.

### 3D short axis image reslicing

To enable a slice-by-slice comparison between 2D- and 3D-LGE images, images corresponding to the 2D SAX images had to be resliced from the 3D data. For multiplanar reslicing of the 3D-CS LGE data a self-developed Python (version 3.7.6) based tool was run in JupyterLab (version 6.0.3). The resliced images from the 3D-LGE data were extracted by using the 2D voxel positions. The in-plane spatial resolution of the 3D-LGE data was degraded in order to reach an equivalent setup as for the reference 2D-LGE. The program to extract equivalent 2D-LGE data from the 3D-LGE dataset worked as follows: The reference 2D-LGE dataset was loaded and the voxel positions with respect to the 3D global coordinate system (GCS) were extracted (ref2Dplane). The 3D voxel positions with respect to the GCS and the corresponding LGE values were read. The ref2Dplane in the GCS was shifted along the surface normal in both directions in steps of 3D-LGE resolution until the distance between the lowest and the highest plane referred to the slice thickness of the 2D-LGE dataset. A linear multidimensional interpolation on regular grids was performed for each 2D plane. The extracted 2D-LGE value in the ref2Dplane was set as the mean across all interpolated 2D plane (ref2Dplane and all shifted planes) LGE values. Consequently, the 2D plane positions reflect the pixel spacing of the 2D-LGE dataset while the mean across the shifted planes refer to an equivalent slice thickness.

### Qualitative and quantitative image analysis

Image analysis was performed by experienced CMR readers (MF, TG, 3- and 5-years experience as an CMR reader), supervised by a cardiac MRI expert (JSM) of 25 years of experience, using CVI42 (Release 5.12.1, Circle Cardiovascular Imaging, Calgary, Canada). Due to the distinctive appearance of 2D- and 3D-LGE images, sequence type was known to the reader but image assessment for 2D and 3D was performed in separate sessions to ensure independent assessment of the corresponding data sets. In case of multiple acquisitions of the same slice, e.g., in order to compensate for motion artifacts, only the final image was evaluated for 2D-LGE. For 2D-LGE magnitude reconstructed images were analyzed as the 3D-CS reconstruction provided magnitude reconstruction only.

#### Subjective and objective image quality

Overall subjective image quality of the 2D and resliced 3D SAX stacks was assessed visually and scored on a four-point Likert scale (0 = poor, non-diagnostic; 1 = fair, diagnostics may be impaired; 2 = good, some artifacts but no interference with diagnostics; 3 = excellent, no artifacts) as described before [22]. The contrast-ratio (CR) between blood pool and myocardium was measured by manually segmenting regions of interest (ROIs) in corresponding basal and apical SAX slices of both 2D and 3D sequences and calculated as

$$CR=\frac{\left| mean\left(Rblood\right) - mean\left(Rmyo\right) \right|}{mean\left(Rblood\right)}$$

where Rblood and Rmyo are the image intensities in the ROIs of the blood pool and normal myocardium, respectively [23].

#### Qualitative fibrosis and scar assessment

All patients were visually examined for the presence of LGE in the myocardium, pericardium, papillary muscles and trabeculae carneae in 2D and 3D images. For 3D-LGE the resliced images as well as the whole 3D dataset were examined.

#### Quantitative fibrosis and scar assessment

**Fig. 1:**
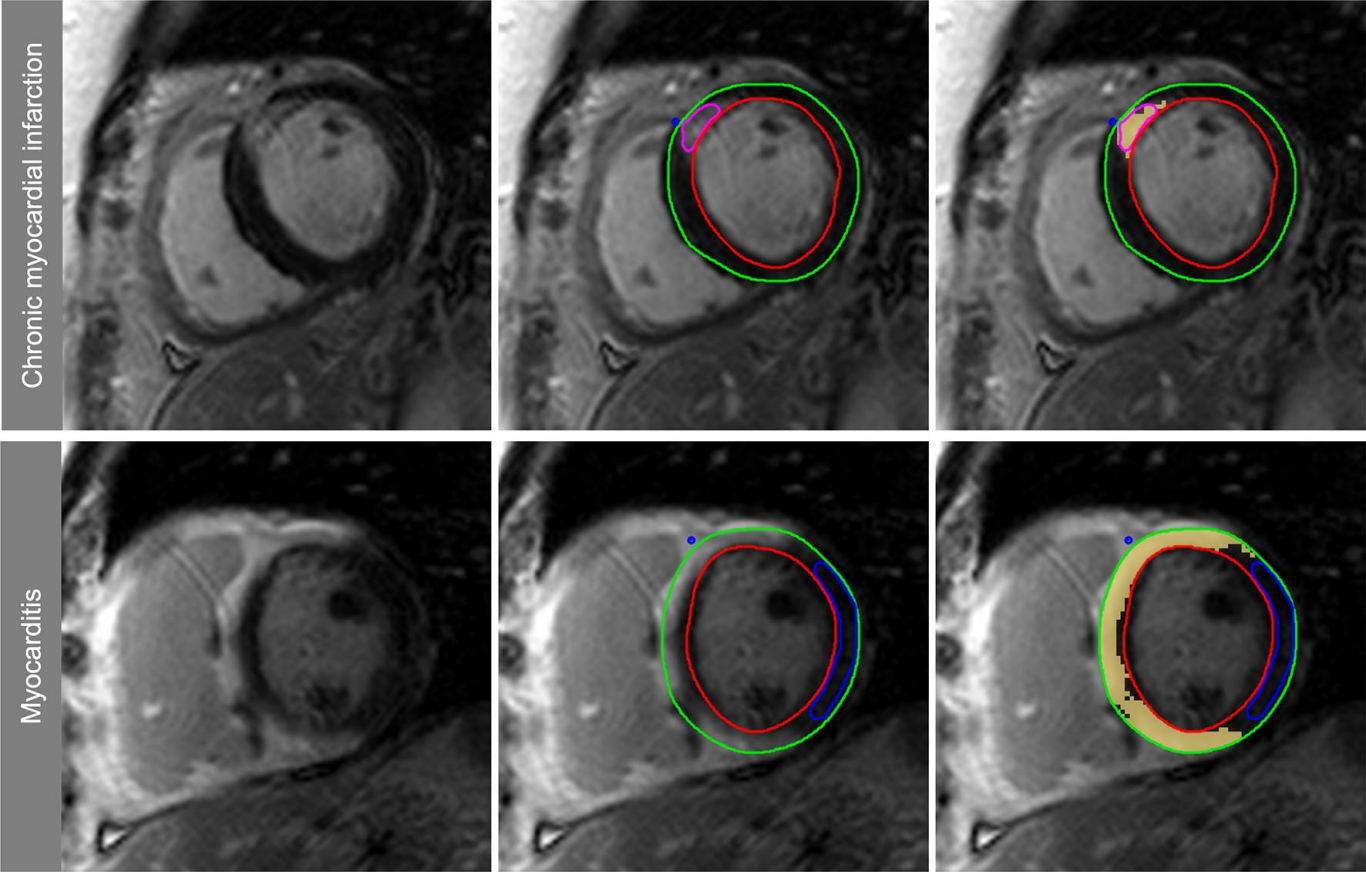
Semiautomatic LGE quantification, endo- (red line) and epicardial (green line) borders were delineated, for quantification of ischemic scars the full-width at half maximum (FWHM) approach was used (upper images), for quantification of LGE in patients with myocarditis we used a mean plus three standard deviation (3-SD) approach. For FWHM the reference region of interest (ROI, pink contour) was drawn into visible scar and covered the region with the visually highest signal intensity. The full width of the myocardial ROI SI histogram at half the maximal signal within the scar served as the threshold between normal myocardium and LGE [26]. For the 3-SD approach the reference ROI (blue contour) was drawn into remote myocardium (defined as no enhancement and normal wall motion), mean signal intensity plus 3 SD in these ROI served as the threshold for the remaining myocardium

Quantitative LGE extent was assessed from the SAX stacks using the semi-automatic full-width at half maximum approach (FWHM) for ischemic fibrosis and scarring [24] and the 3-standard deviations (SD) thresholding approach for MYC [25] accounting for the heterogeneity in scar-signal intensities between these disease entities (see Fig. 1). Endo- and epicardial boarders were drawn manually according to SCMR guidelines [26]. Biplane LVEF and volume parameters were calculated automatically by the post-processing software according to an in-line biplane ellipsoid model [27]. Long-axis LGE images as well as cine images were provided to the reader for reference. ROIs for thresholding were drawn in every slice to obtain suitable segmentation and to account for external factors (image noise, inversion time, surface coil intensity variations). If no enhancement was found in a slice, the maximum signal of the nearest slice with enhancement was used for the FWHM approach and if a slice contained no remote normal myocardium, mean and SD of the nearest slice with normal remote myocardium was used for the 3-SD approach [28]. The automated LGE detection could be manually corrected to account for obvious artifacts, e.g., motion artifacts, partial volume effects, or artifacts caused by epicardial fat. Myocardial mass, scar tissue mass (in grams, g) and LGE extent (in %) were calculated for each patient and sequence. Distribution area and extent of fibrosis within a segment were evaluated according to the American Heart Association (AHA) 16-segment model and LGE extent per segment was grouped in 5 categories (no LGE = 0; 1–25% = 1; 26–50% = 2; 51–75% = 3; 76%-100% = 4).

#### Inter and intra-rater agreement

For 10 patients with CMI a second experienced reader (TG) and the first reader (MF) repeated LGE quantification. Cases were selected randomly from different image quality categories to ensure equal representation.

### Statistical analysis

All statistical analyses were conducted using statistical software packages GraphPad Prism Version 9.1.0 (GraphPad Software, LLC, San Diego, CA, USA) and SAS Version 9.4 (SAS Institute Inc., Cary, NC, USA). Figures were generated in GraphPad Prism and Microsoft Excel Version 14.7.7 (Microsoft Cooperation, Redmond, WA, USA). Statistical comparison of myocardial mass, scar mass, and LGE extent between 2D and 3D was performed by Student’s paired two-tailed *t*-tests. Bland–Altman plots were generated to assess bias (mean difference) and 95% limits of agreement for each parameter. Correlation between 2D and 3D was assessed using Pearson correlation coefficient. Subjective image quality scores and acquisition time were compared using Wilcoxon matched-pairs signed-rank test and Friedman’s test with Dunn’s test for post-hoc comparison was used to compare CR between sequences and regions. Fisher’s exact test was used for comparison of the distribution of categorical variables and McNemar’s test to compare 2D and 3D sequence regarding ability to detect papillary and trabecular infarction.

Inter-observer agreement on myocardial mass, scar mass and LGE extent was determined using Pearson correlation coefficient. Multinomial regression within a mixed model with repeated measures was used to assess differences in group allocation of LGE extent per segment (groups 0 to 4) using compound symmetry to model the correlation between up to 16 segments within the same patient. Level of significance was set to α = 0.05.

## Results

### Patient characteristics and image acquisition

3D-CS was acquired in all patients. Patients with MYC were significantly younger, had a lower body mass index (BMI), higher LVEF and lower LVM-Index (Table 1).

**Table 1:** Patient characteristics

	Chronic myocardial infarction	Myocarditis	*p*
Number of patients	33	20	/
Gender (male/female)	25/8 (75.8%/24.2%)	16/4 (80.0%/20.0%)	> 0.99
Age (years)	63.3 ± 13.4	42.2 ± 13.4	< 0.01
BMI (kg/m^2^)	27.1 ± 3.3	25.3 ± 5.0	< 0.05
HR (min^−1^)	69.0 ± 12.3	72.5 ± 17.5	0.39
LVEF (%)	50.9 ± 11.1	55.6 ± 10.5	< 0.05
LVEDV-I (ml/m^2^)	89.4 ± 19.1	90.2 ± 18.2	0.88
SV-I (ml/m^2^)	44.7 ± 11.2	50.1 ± 11.6	0.09
LVM-I (g/m^2^)	65.0 ± 13.4	56.6 ± 11.6	< 0.01

*BMI* body mass index, *HR* heart rate, *LVEF* left ventricular ejection fraction, *LVEDV-I* left ventricular end-diastolic volume index, *SV-I* stroke volume index, *LVM-I* left ventricular mass index

In 39.6% of scans (21 of 53; CMI: 15 of 33, MYC: 6 of 20) 3D images were obtained before 2D images. Mean time between contrast agent application and start of image acquisition were 14:47 (2D-LGE) and 16:10 (3D-LGE) minutes (*p* = 0.58), respectively. Because of poor 2D image quality caused by impaired bh capability, additional free-breathing 2D-PSIR MOCO imaging was acquired in 11 patients (5 with CMI, 6 with MYC). Data from 3 patients with MYC were excluded from quantitative LGE assessment due to non-diagnostic image quality in 3D images. Reasons for impaired image quality in these patients were loss of ECG-trigger signal (*n* = 1), tachycardia (*n* = 1) and heavy thorax motion caused by shortness of breath due to heart failure (*n* = 1). Another dataset from the MYC group was excluded from LGE assessment because no LGE was detected on either 2D or 3D images.

### Acquisition time

**Fig. 2:**
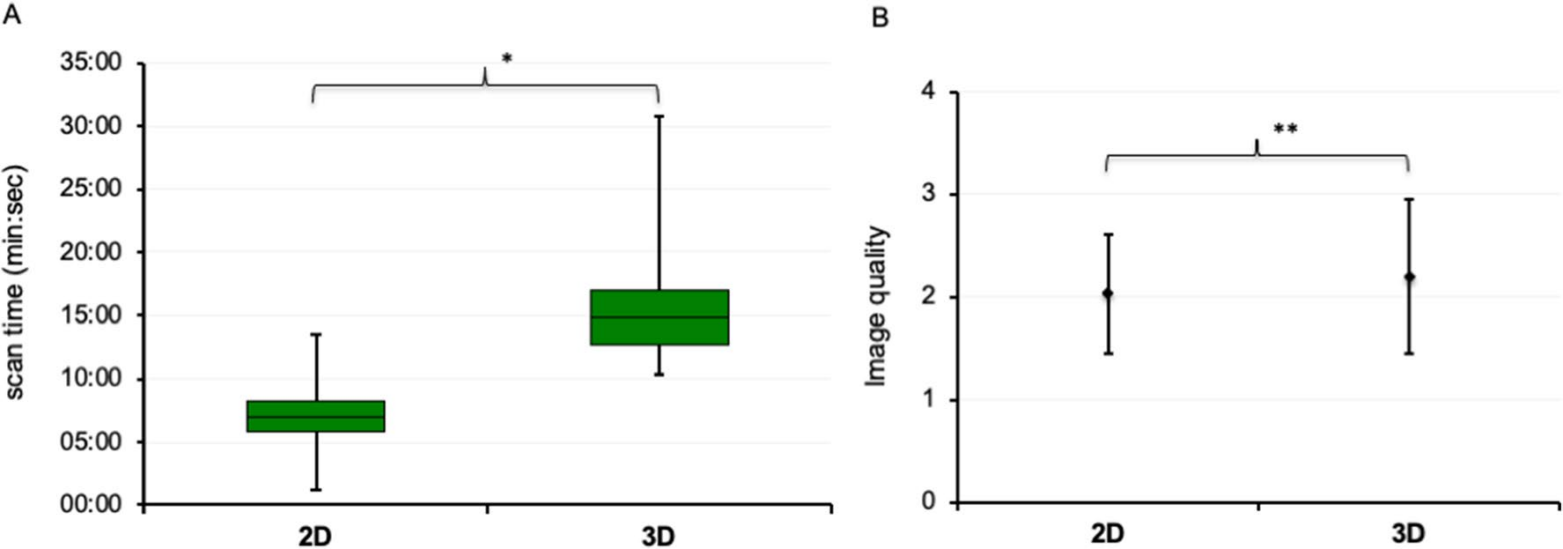
Acquisition time and subjective image quality. **A** Boxplot for acquisition time (min:sec), *p < 0.01. **B** Subjective image quality; values represent mean ± SD, score system: 3 = excellent quality, no artifacts; 2 = good quality, minimal artifacts; 1 = moderate quality, some artifacts which may impair diagnostic quality; 0 = poor quality, non-diagnostic; **p < 0.05

The average acquisition time for the 2D sequence compared to the 3D sequence was significantly shorter (2D: median: 06:59 min [IQR: 05:51–08:18]; 3D: 14:48 min [12:45–16:57], see Fig. 2) while no difference between 2D PSIR and 2D MOCO LGE could be observed (*p* = 0.08).

### Image quality

2D- and 3D-LGE sequences offered both good to excellent image quality in > 85% of patients (2D-LGE: 87.8%; 3D-LGE: 91.8%) but mean subjective image quality score was significantly better for 3D-LGE (2D-LGE: 2.06 ± 0.56; 3D-LGE: 2.29 ± 0.61; *p* < 0.05). Main reasons for impaired image quality for 2D-LGE were motion artifacts, presumably caused by impaired breath-holding. CR was significantly lower in 3D-CS basal and apical regions than in corresponding 2D-LGE regions (CR basal: 2D: 0.77 ± 0.11, 3D: 0.59 ± 0.10, *p* < 0.01; CR apical: 2D: 0.64 ± 0.17, 3D: 0.53 ± 0.11, *p* < 0.05). CR did not differ significantly between basal and apical regions in images acquired with 3D-CS (*p* = 0.63), whereas CR was significantly higher in 2D basal regions compared to 2D apical regions (*p* < 0.01).

### Qualitative scar assessment

**Fig. 3:**
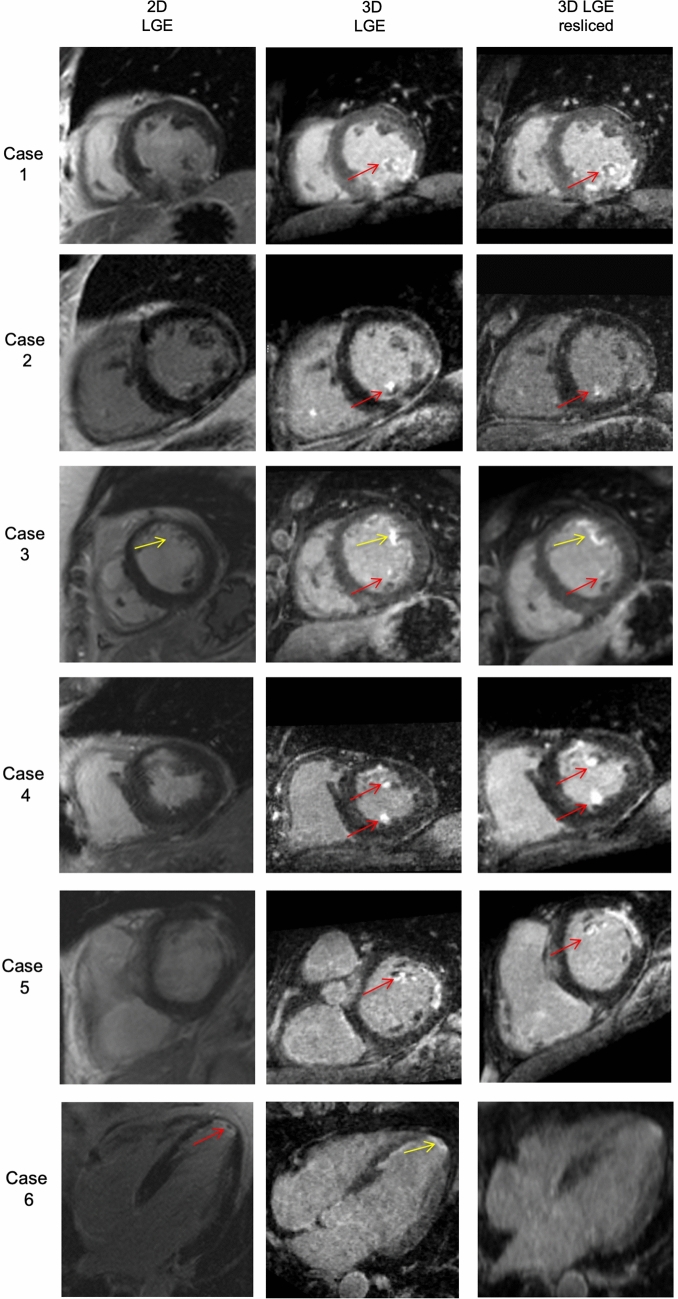
Divergent findings between 2D- and 3D-LGE sequences, Case 1: Chronic myocardial infarction (CMI) inferior and inferolateral, incomplete infarction of the inferior papillary muscle was present only in 3D images (red arrow); Case 2: CMI inferolateral, incomplete infarction of the inferior papillary muscle was present only in 3D images (red arrow); Case 3: CMI anterior, incomplete infarction of the anterior papillary muscle was present in 2D and 3D images (yellow arrow), infarction of the posterior papillary muscle was present only in 3D images (red arrow); Case 4: CMI anterior, incomplete infarction of the anterior papillary muscle and inferior trabeculae was present in 3D images only (red arrows); Case 5: CMI anterolateral, incomplete infarction of the anterior papillary muscle was present only in 3D images (red arrow); Case 6: CMI midventricular septal and apical circumferential, apical thrombus (3.8 × 2.6 mm) was clearly present only in 2D images (red arrow), not visible in resliced 3D images and scarcely visible in high resolution 3D-LGE images; “3D-LGE” short axis view (SAX) exported from 3D dataset after manual multiplanar reformatting, resolution = 1.25^3^ mm^3^, 3D-LGE resliced = SAX view extracted from 3D dataset corresponding to 2D image by using the 2D voxel positions, in-plane resolution and slice thickness was set to match 2D resolution (1.4 × 1.4 mm^2^, slice thickness = 7 mm)

Myocardial LGE was visually detected in all included patients. There were no cases in which LV-myocardial LGE was present in one sequence and could not be detected in the corresponding image acquired with the other sequence. Papillary or trabecular infarction was detected in 11/33 CMI patients using the 2D-LGE sequence while the 3D dataset—especially after using multiplanar reformation—allowed clear identification in 16/33 CMI patients (*p* = 0.07; in 2/5 of these divergent cases 2D sequence was acquired first, see Fig. 3. An apical left ventricular thrombus (3.8 × 2.6 mm) could be detected in one patient using 2D-LGE images but was scarcely visible in 3D-LGE images (Fig. 3). In this case 2D-LGE images were obtained first (04:17 min after contrast application), 3D-LGE-image acquisition was started 12:28 min after contrast application and acquisition time was 15:42 min.

### Quantitative scar assessment

**Fig. 4:**
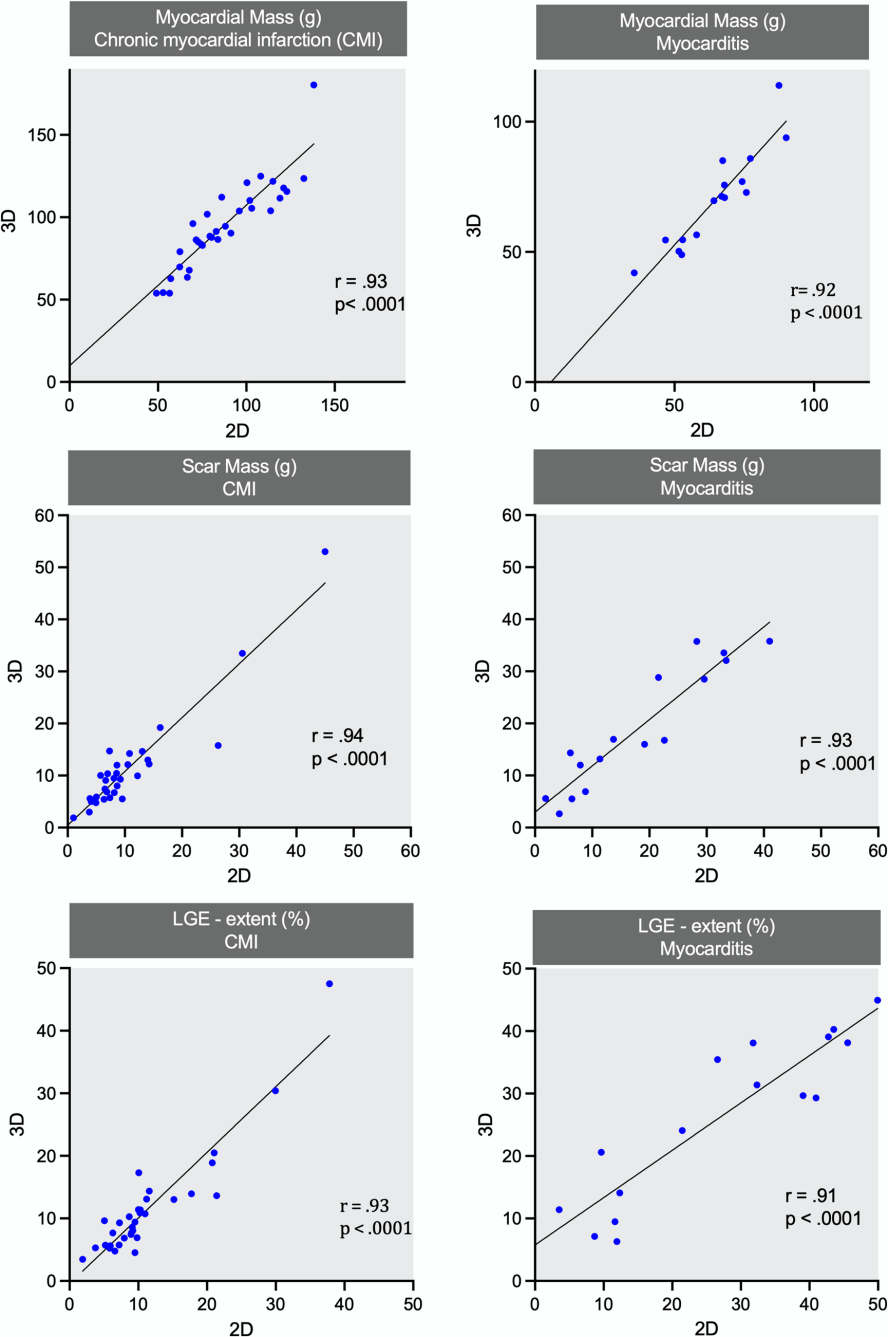
Correlation between 2D- and 3D-LGE sequences regarding myocardial mass (g), scar mass (g) and LGE extent (%)

**Fig. 5:**
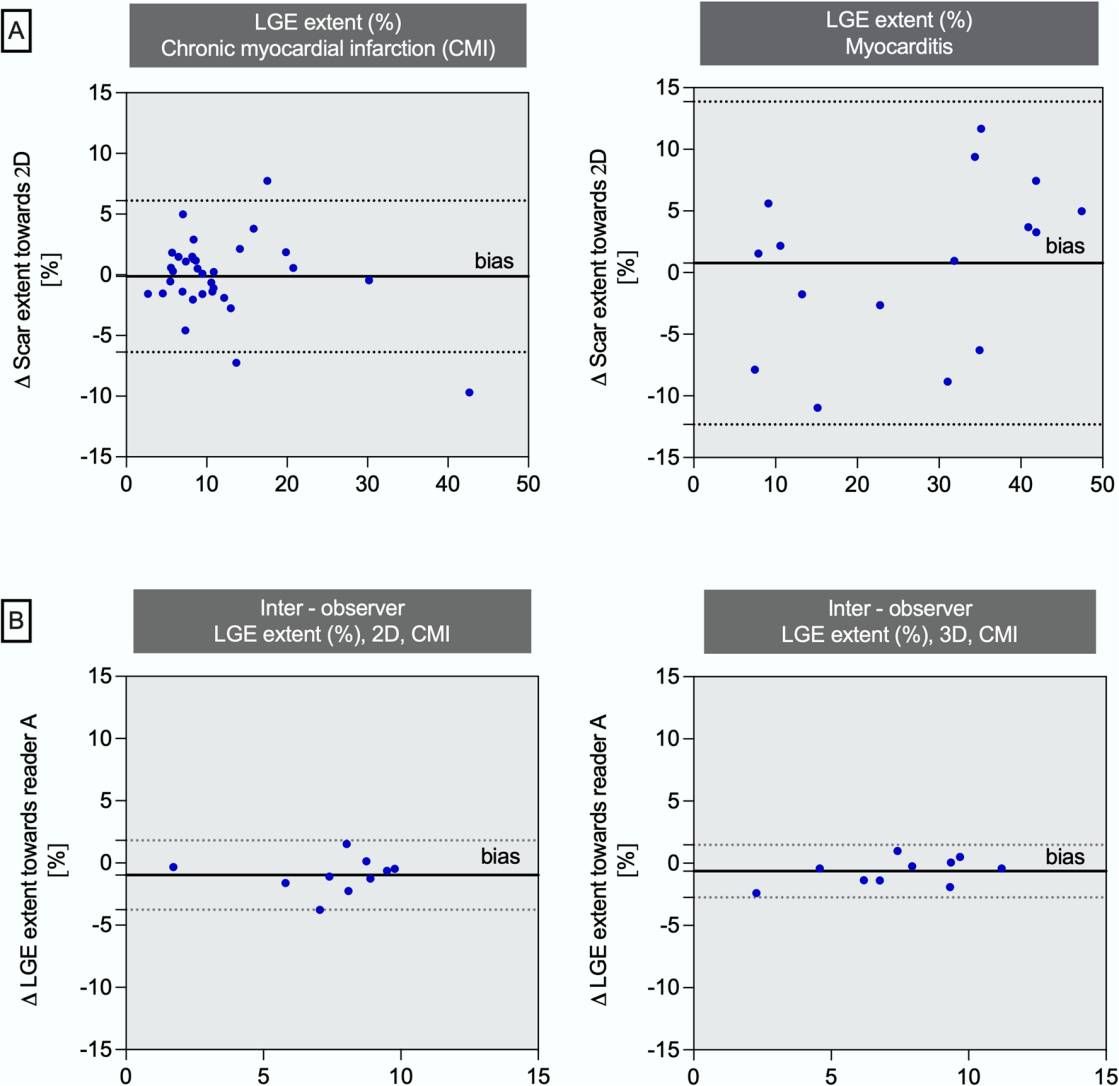
**A** Bland Altman plots of LGE extent (%) for agreement between 2D and 3D sequences. Blue dots represent mean between 2D- and 3D-LGE (x-axis) versus delta towards 2D (y-axis); **B** Bland Altman plots of LGE extent (%) for agreement between reader A and reader B. Blue dots represent mean between 2D- and 3D-LGE (x-axis) versus delta towards reader A (y-axis)

**Fig. 6:**
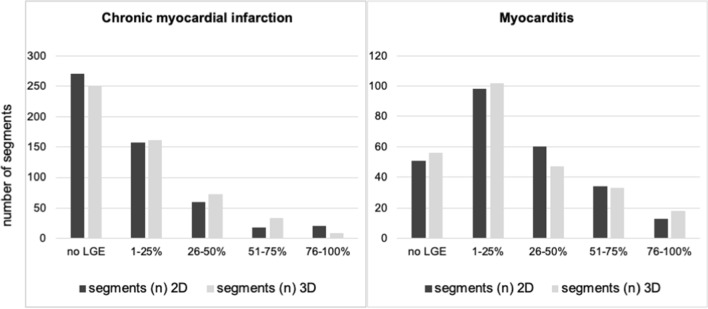
Quantitative assessment of LGE-extent per segment, y-axes represent total number of AHA-segments, x-axes represent LGE-extent (%) categories

The correlation between 2 and 3D-CS regarding overall myocardial mass, scar mass and LGE extent was substantial (*r* > 0.91) and statistically reliable in patients with CMI and MYC (Fig. 4). There was no significant difference between 2D- and 3D-LGE groups regarding global LGE extent (Fig. 5). We did not find a significant difference in group allocation of LGE extent (0 to 4) per segment between 2D and 3D sequence (*p* = 0.63; see Fig. 6 for absolute number of segments within LGE extent categories). 3D-LGE significantly overestimated myocardial mass in both disease entities and showed a trend towards overestimation of scar mass in CMI; however, no bias was found in the assessment of LGE extent (Table 2).

**Table 2:** Quantitative assessment of myocardial mass, scar mass, and LGE extent

	2D-LGE	3D-LGE	Bias ± SD	*r*
CMI				
Global myocardial mass (g)	87.3 ± 24.1	94.9 ± 26.1	– 7.6 ± 11.7 (*p* < 0.01)	0.93 (*p* < 0.01)
Global scar mass (g)	10.3 ± 8.7	11.1 ± 9.5	– 0.8 ± 3.2 (*p* = 0.06)	0.94 (*p* < 0.01)
Global LGE extent (%)	11.4 ± 7.5	11.5 ± 8.5	– 0.1 ± 3.2 (*p* = 0.99)	0.93 (*p* < 0.01)
MYC				
Global myocardial mass (g)	64.7 ± 14.7	70.2 ± 18.9	– 5.4 ± 7.7 (*p* < 0.05)	0.92 (*p* < 0.01)
Global scar mass (g)	18.1 ± 12.2	19.0 ± 11.6	– 0.9 ± 4.4 (*p* = 0.40)	0.93 (*p* < 0.01)
Global LGE extent (%)	27.0 ± 15.7	26.2 ± 13.1	0.8 ± 6.7 (*p* = 0.70)	0.91 (*p* < 0.01)

*CMI* chronic myocardial infarction, *MYC* Myocarditis, *LGE* late gadolinium enhancement, *r* Pearson correlation coefficient between 2D-LGE and 3D-LGE

**Fig. 7:**
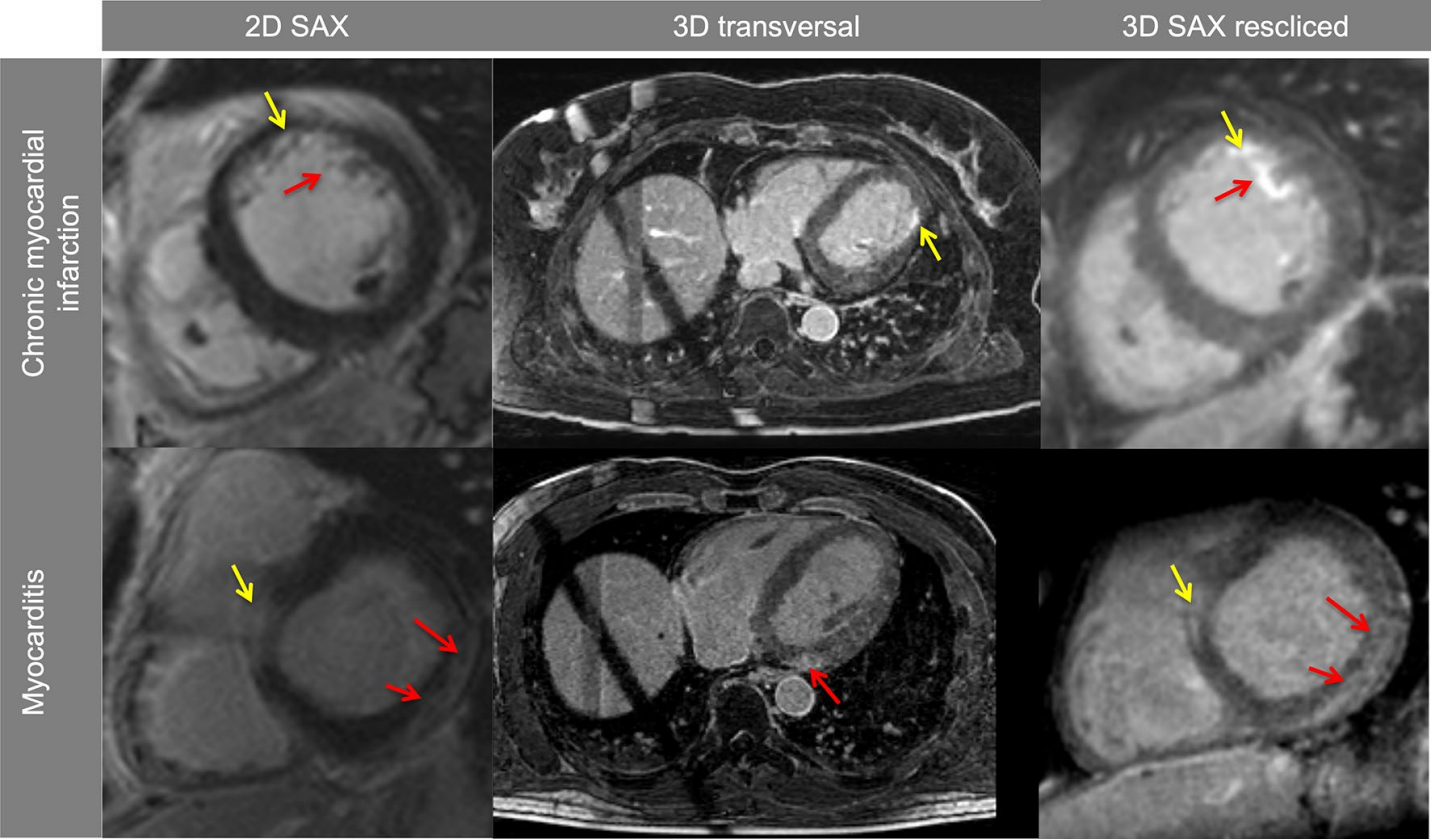
Typical case examples for chronic myocardial infarction (CMI) and myocarditis; CMI: Midventricular anterior myocardial (yellow arrow) and trabecular (red arrow) infarction, derived from the same patient and slice location; Myocarditis: Basal inferior and lateral subepicardial enhancement (red arrows), derived from the same patient and slice location, yellow arrow indicates left ventricular outflow tract; *SAX* short axes view

**Fig. 8:**
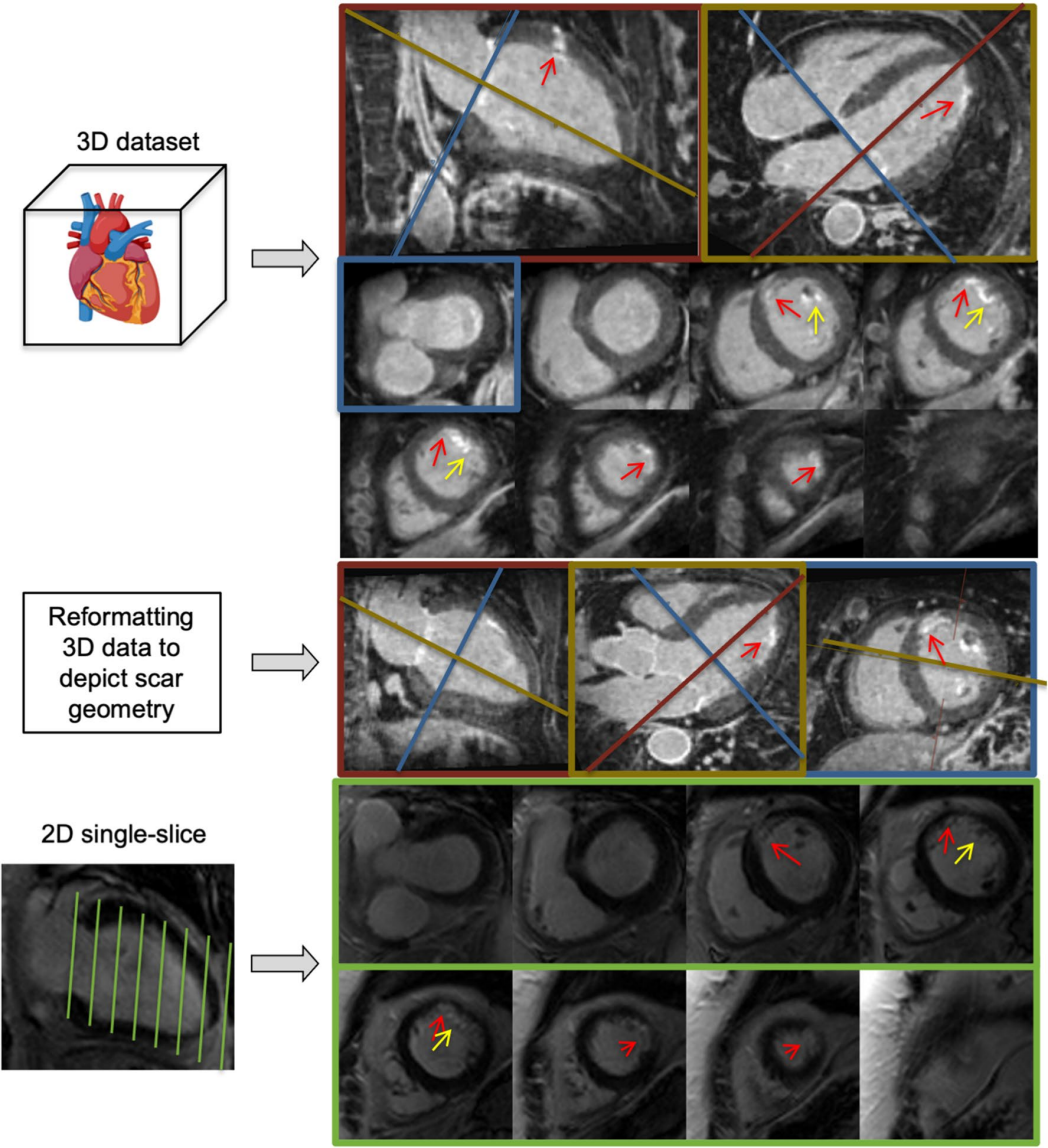
3D-CS offers whole heart coverage in one acquisition, enabling multiplanar reformatting to depict scar geometry. Here, 4-Chamber view (CV), 2-CV, 3-CV and short axes views (SAX) are shown as examples. For 2D scar assessment, multiple 2D SAX slices must be acquired successively to cover the left ventricle. Red arrows indicate subendocardial scarring. Yellow arrows indicate trabecular infarction

Pearson coefficient indicated a high inter- and intra-observer correlation regarding myocardial mass, scar mass and LGE extent in both sequences (inter-observer: myocardial mass 2D: *r* = 0.96; scar mass 2D: *r* = 0.89; LGE extent 2D: *r* = 0.83; myocardial mass 3D: *r* = 0.90; scar mass 3D: *r* = 0.97; LGE extent 3D: *r* = 0.94; all *p*s < 0.01; intra-observer: myocardial mass 2D: *r* = 0.99; scar mass 2D: *r* = 0.94; LGE extent 2D: *r* = 0.89; myocardial mass 3D: *r* = 0.90; scar mass 3D: *r* = 0.88; LGE extent 3D: *r* = 0.86; all *p*s < 0.01). The intra-reader bias with respect to LGE extent (%) was 1.15 in the 2D and 1.97 in the 3D sequence, respectively. Bland Altman analysis demonstrated small bias between both readers (Fig. 5). Typical case examples are provided in Figs. 7 and 8.

## Discussion

The present study compared a non-breath-hold 3D-CS prototype LGE sequence with high spatial resolution and isotropic voxel size to a 2D-LGE reference standard regarding image quality and LV fibrosis quantification in patients with ischemic and non-ischemic heart disease. Our main findings were: there was no significant quantitative difference between novel 3D-CS and routine 2D-LGE imaging in the assessment of scar extent. 3D-CS obtained better subjective quality scores. The acquisition time for the 3D-CS sequence was significantly longer. In five cases papillary or trabecular infarction was present in 3D but not in 2D images. Taken together, it indicates usability and high potential of this novel 3D technique for clinical use in selected patient cohorts to delineate smaller cardiac structures and pathologies. However, the addition of PSIR reconstruction and reduction of acquisition time is warranted.

CMR using 2D-LGE is the long-established method for the determination of left ventricular myocardial scarring in patients with ischemic and non-ischemic cardiomyopathies, but isotropic 3D-LGE sequences are increasingly used [13, 16, 29] due to their potential advantages, for example, whole heart coverage in a single scan, better depiction of complex scar geometry, detection of pathologies in smaller cardiac structures, and identification of peri-infarct zones [30]. The equivalent quantitative detection of left ventricular scar extent in patients with CMI and MYC in our study is in agreement with the results of Pennig and colleagues who visually found no difference in global LGE between a 2D-LGE sequence and a compressed SENSE accelerated 3D high isotropic resolution LGE sequence in patients with ischemic and non-ischemic heart disease [13]. In addition, the 3D-CS sequence allowed detection of small LGE findings (papillary and trabecular infarction) in 5 cases in which the 2D sequence did not show LGE appearance. On the other hand, in one case an apical thrombus could scarcely be detected using the 3D-CS sequence. While the better detection of small findings probably can be attributed to the higher isotropic resolution, we hypothesize that the thrombus was scarcely visible due to contrast agent inflow into the thrombus material at the time of 3D-CS imaging.

However, despite showing comparable scar extent, 3D-CS overestimated myocardial mass and showed a trend towards higher scar mass in our study. Bizino and colleagues compared reformatted “normal” resolution (1.46 × 1.46 × 10 mm^3^) and “high” resolution 3D-LGE images (isotropic 0.91 mm^3^) derived from the same 3D-LGE dataset obtained in free-breathing technique at 3 Tesla [31]. “Normal” resolution images significantly overestimated scar mass—a finding which was attributed to better scar border delineation and less partial volumes effects in higher resolution images. However, we intentionally matched the 2D spatial resolution when reslicing the 3D images to avoid differences in partial volumes effects. In our study, the trigger delay for 3D-CS was determined visually based on the least motion phase of diastole according to cine images. The acquisition of images in a phase more shifted to systole may have led to an increase in myocardial and scar mass.

The 3D-CS sequence obtained better subjective image quality scores even though the 2D-LGE reference standard already provided good to excellent image quality ratings in > 85% of patients. This finding is in line with recent publications [13, 16] in which readers graded scans with good to excellent image quality in > 80% of CS accelerated 3D-LGE scans [13]. Importantly, this also holds true albeit identical 3D slices were reconstructed according to 2D-LGE SAX slices to allow unbiased comparison, i.e., with higher slice thickness. Thus, potential advantages of the 3D sequence were limited as the high isotropic resolution was not fully exploited, e.g., as shown for trabecular infarction (Figs. 3 and 7).

Recently, addressing reduced image quality caused by impaired breath-holding capabilities, a free-breathing MOCO 2D-LGE sequence was introduced demonstrating better image quality and reduced acquisition time when compared to 2D bh LGE [9]. Nonetheless, spatial resolution of the MOCO LGE sequence was 1.4 × 1.9 mm^2^ (8 mm slice thickness), once again exceeding certain anatomical dimensions and potentially causing partial volume effects by using anisotropic voxel sizing.

The acquisition time of the 3D sequence amounted to approximately twice the acquisition time of the 2D sequence with a maximum duration of 30:47 min in one extreme case caused by a low acceptance rate of navigator-based gating due to irregular breathing; nonetheless overall acquisition time lay within an acceptable time window for infarct size detection [32].

Thus, there seems to be a trade-off between LGE image quality, spatial resolution and acquisition time. Furthermore, the 3D-LGE sequence does not offer PSIR reconstruction which is less sensitive to unprecise myocardial nulling [33]. These factors may constitute an important aspect of the decision to incorporate this sequence into clinical routine. However, it must be considered that the 3D-CS sequence offers whole heart coverage with high spatial resolution while only left ventricular coverage was accomplished with conventional 2D-LGE. Covering the atria using 2D-LGE with SAX slices (7 mm slice thickness, no gap) takes another 3–7 min depending on the size of the atria. Accordingly, the 3D-CS approach offers a highly promising tool for a more comprehensive cardiovascular risk stratification. Further reduction of the scan time and introduction of PSIR reconstruction is addressed in new developments as, for example, a recently developed isotropic 3D fat-water LGE sequence deploys direct navigator based respiratory motion tracking of the heart and non-rigid motion correction overcoming low acceptance rates and offering PSIR reconstruction [29, 34]. Another approach to reduce acquisition time is to use non-cartesian acquisition schemes. The incorporation of a non-selective inversion pulse, a 3D radial SSFP read-out scheme and respiratory self-navigation yielded faster image acquisition compared with standard 2D-LGE [35].

Moreover, CR did not differ significantly between basal and apical regions in 3D-CS images, whereas CR did decrease significantly in 2D apical images. This is most likely due to randomly scanning the whole volume with one set of parameters integrated over time. Importantly, this allows consistent fibrosis quantification across the whole volume without adapting thresholds slice-wise, e.g., for scar intensity or normal myocardium. Even though the examined 3D sequence lacks implementation of a PSIR reconstruction and a reduction of acquisition time is warranted before wider application in clinical routine, advanced 3D segmentation methods may be applied more straight forward for accurate assessment of fibrosis in the future. The data presented in our study show high congruency between isotropic 3D-CS LGE and standard magnitude 2D images. Furthermore 3D-LGE enabled the detection of pathologies in thin structures. Therefore, the high isotropic resolution may allow accurate delineation and quantification of fibrosis in thin cardiac structures, i.e., the atria and right ventricle, thus offering high potential for a more comprehensive cardiovascular risk stratification which should be addressed in future studies.

## Limitations

A noise scan for accurate noise estimation should be obtained in studies assessing 3D-LGE accelerated parallel imaging sequences as discussed by Bratis and colleagues [36]. Due to the integration of the study into clinical routine, we were not able to implement noise scans for time reasons. Due to logistic problems, randomization of acquisition order did not work in 9.4% cases (5/53). MOCO 2D-LGE imaging was not performed in all participants because it was not available to us at study start. The results of this study may not be generalizable to populations with other LGE appearances (e.g., HCM, DCM, Amyloidosis). Comparability between 3D-CS LGE and standard 2D-LGE in these entities should be investigated in future studies. The longer acquisition time observed with the 3D sequence may lead to discomfort in certain patient groups (e.g., patients with decompensated heart failure).

## Abbreviations

3-SD: Three-standard deviations
AHA: American Heart Association
bh: Breath- hold
bSSFP: Balanced steady state free precession
CMI: Chronic myocardial infarction
CR: Contrast-ratio
CS: Compressed sensing
CV: Chamber view
ECG: Electrocardiography
EDV: End diastolic volume
FA: Flip angle
FISTA: Fast iterative shrinkage thresholding algorithm
FOV: Field of view
FWHM: Full-width at half maximum
GRE: Gradient echo
HCM: Hypertrophic cardiomyopathy
IR: Inversion recovery
LGE: Late gadolinium enhancement
LV: Left ventricle
LVEF: Left ventricular ejection fraction
LVM: Left ventricular mass
MOCO: Motion corrected
MYC: Myocarditis
PSIR: Phase sensitive inversion recovery
ROI: Region of interest
TE: Echo time
TI: Inversion time
TR: Repetition time
